# DNA methylation differences between *in vitro*- and *in vivo*-conceived children are associated with ART procedures rather than infertility

**DOI:** 10.1186/s13148-015-0071-7

**Published:** 2015-04-08

**Authors:** Sisi Song, Jayashri Ghosh, Monica Mainigi, Nahid Turan, Rachel Weinerman, May Truongcao, Christos Coutifaris, Carmen Sapienza

**Affiliations:** Fels Institute for Cancer Research and Molecular Biology, Temple University School of Medicine, 3307 N Broad Street, Philadelphia, PA 19140 USA; Department of Obstetrics and Gynecology, University of Pennsylvania School of Medicine, 3701 Market Street, 8th Floor, Philadelphia, PA 19119 USA; Department of Pathology and Laboratory Medicine, Temple University School of Medicine, Philadelphia, PA 19140 USA

**Keywords:** DNA methylation, Assisted reproduction, Donor oocytes, Infertility, Placenta

## Abstract

**Background:**

We, and others, have demonstrated previously that there are differences in DNA methylation and transcript levels of a number of genes in cord blood and placenta between children conceived using assisted reproductive technologies (ART) and children conceived *in vivo*. The source of these differences (the effect of ART *versus* the underlying infertility) has never been determined in humans. In this study, we have attempted to resolve this issue by comparing placental DNA methylation levels at 37 CpG sites in 16 previously identified candidate genes in independent populations of children conceived *in vivo* (‘fertile control’ group) with ART children conceived from two groups: either autologous oocytes with infertility in one or both parents (‘infertile ART’ group) or donor oocytes (obtained from young fertile donors) without male infertility (‘donor oocyte ART’ group).

**Results:**

Of the 37 CpG sites analyzed, significant differences between the three groups were found in 11 CpGs (29.73 %), using ANOVA. Tukey’s *post hoc* test on the significant results indicated that seven (63.63 %) of these differences were significant between the donor oocyte ART and fertile control groups. In addition, 20 of the 37 CpGs analyzed had been identified as differentially methylated between ART and fertile control groups in an independent population in a prior study. Of these 20 CpG sites, 9 also showed significant differences in the present population. An additional 9 CpGs were found to be significantly different between the two groups. Of these 18 candidate CpGs, 12 CpGs (in seven candidate genes) also showed significant differences in placental DNA methylation levels between the donor oocyte ART and fertile control groups.

**Conclusions:**

These data suggest strongly that the DNA methylation differences observed between ART and *in vivo* conceptions are associated with some aspect of ART protocols, not simply the underlying infertility.

**Electronic supplementary material:**

The online version of this article (doi:10.1186/s13148-015-0071-7) contains supplementary material, which is available to authorized users.

## Background

Modest but significant differences in DNA methylation have been identified between children conceived *in vivo* and children conceived by assisted reproductive technology (ART) [1-10]. While there are some inconsistencies between studies [11-20], it seems likely that these differences in methylation between these two groups are genuine. This conclusion is supported by animal studies which find that ART leads to epigenetic changes in offspring [21-29]. At this juncture and given these observations, there are at least two important questions to be answered: 1) Are epigenetic differences in ART pregnancies associated with the observed increase in adverse outcomes in these pregnancies and 2) are the ART-associated differences the result of clinical and/or laboratory interventions used in ART or are they associated with the diagnosis of infertility itself?

In a recent study, we began to address the first question, that is, whether ART-associated epigenetic differences are associated with adverse outcomes by demonstrating that DNA methylation levels at a modest number of loci can explain a large fraction of variance in infant birth weight [30]. It is critical to note that low birth weight (LBW) is the most significant ART-associated adverse perinatal outcome, in terms of number of cases [31-35]. Others have also found that ART is associated with not only adverse neonatal outcomes, such as birth defects, but also long-term outcomes, including growth, gonadal development, physical health, neurological or neurodevelopmental defects and, especially, epigenetic abnormalities [36-52]. Children conceived *in vitro* are generally at higher risk of small for gestational age (SGA), preterm delivery, perinatal morbidity, and hospital admission than children conceived *in vivo* [36,37]. In addition, meta-analyses have revealed a 30% to 40% increase in major malformation rates for infants born after ART compared with naturally conceived children [38-40], and children conceived *via in vitro* fertilization (IVF), who rapidly gained weight during early childhood (1 to 3 years), have been shown to have higher blood pressure levels [41]. Interestingly, even apparently healthy ART-conceived children may have an increased risk of cardiovascular diseases later in life [42-45]. Finally, publications from Europe, the United States, and Australia have all suggested an association between ART and imprinting disorders such as Beckwith-Wiedemann syndrome [37,46-52]. As the number of children born following ART continues to increase, it is critical to understand whether epigenetic changes are responsible for some of the adverse outcomes observed following ART.

In the current study, we began to address the question of whether ART-associated differences in DNA methylation are a characteristic of ART-related protocols and/or procedures (clinical or laboratory) or the diagnosis of infertility, *per se*, by utilizing a group of patients undergoing ART with no infertility diagnosis. In this study, we utilized bisulfite pyrosequencing to analyze placental DNA methylation of genes previously identified by our laboratory as being differentially methylated between *in vitro*- and *in vivo*-conceived children using array-based methods. We compared placental DNA methylation in children conceived *in vivo* (fertile control group) with ART children conceived following fertilization of oocytes from two distinct groups: 1) autologous oocytes with infertility in one or both parents (infertile group); 2) donor oocytes (obtained from young fertile donors) without male factor infertility in the recipient’s partner (donor oocyte group). It is clear that the oocytes, sperm, and the resulting embryos in the donor group, which were obtained from patients without infertility diagnoses, are still subjected to the clinical and laboratory procedures of ART. Therefore, such comparison of the infertile and donor groups should distinguish DNA methylation differences that are the result of ART protocols and/or procedures from effects caused by an infertility diagnosis.

## Results

The demographic, cycle, and birth characteristics of the fertile control, infertile ART, and donor oocyte ART groups are shown in Table 1. Mean maternal age, paternal age, gestational age, and birth weight differed significantly between the groups, as expected. Intra-cytoplasmic sperm injection (ICSI) was utilized in 13.6% of the cycles in the infertile ART group for insemination, while ICSI was not used in any of the donor cycles. The placental DNA methylation levels were analyzed by bisulfite conversion and pyrosequencing at 37 candidate CpG sites in 16 genes. These genes were selected based on previous array studies in our laboratory (see ‘Methods’ for details of molecular analysis and CpG site selection). One-way analysis of variance (ANOVA) was used to assess differences between the control, infertile, and donor groups, followed by Tukey’s *post hoc* honest significant difference (HSD) test. The secondary goal of this study was to validate our previous findings of differences in placental DNA methylation between *in vivo*- and *in vitro*-conceived children. We had previously identified differences in 20 of the targeted CpG sites in 15 genes [30]. We therefore compared placental DNA methylation between the fertile control and infertile groups in these 15 genes for independent validation using a one-tailed *t*-test. Finally, we tested the differences between the donor oocyte ART and fertile control groups at the sites for which differences between the control group and the infertile group existed. The differences between the fertile control group and donor oocyte ART group were tested using a two-tailed *t*-test.

**Table 1:** **Demographic and relevant clinical data for subjects**

	**Infertile ART group (** ***n*** **= 66)**	**Donor oocyte ART group (** ***n*** **= 22)**	**Fertile control group (** ***n*** **= 49)**	***P*** **-value** ^**a,b**^
Maternal age (years, mean ± SD)	35.3 ± 3.7	41.5 ± 6.0^c^	34.5 ± 5.0	*<0.0001*
Paternal age (years, mean ± SD)	36.2 ± 5.3	42.4 ± 7.1	34.9 ± 5.7	*<0.0001*
IVF cycle type (fresh/frozen)	58/8	18/6	Not applicable	0.1871^d^
Number of ICSI cycles	9 (13.6 %)	0 (0 %)	Not applicable	0.1047^d^
Gestational age (weeks, mean ± SD)	38.8 ± 1.9	37.7 ± 2.3	39.3 ± 1.0	*0.0014*
Birth weight (grams, mean ± SD)	3,371 ± 625	3,079.7 ± 636	3,640 ± 459	*0.0007*
Males (%)	32 (48.5 %)	9 (40.1 %)	25 (51.0 %)	0.7310^d^
Females (%)	34 (51.5 %)	13 (59.0 %)	24 (49.0 %)	

^a^ *P*-values from ANOVA unless indicated; ^b^values in italics denote significance;^c^mean age of the recipient. All donors were fertile women between the ages 21 and 33; ^d^ *P*-value from chi-square. *n* = number of placentae.

**Figure 1:**
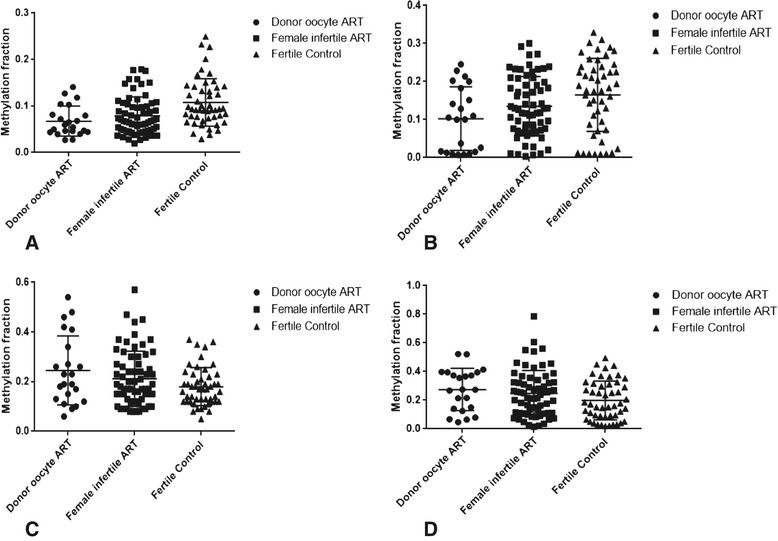
**Graphs of methylation fractions in different groups- donor oocyte ART, infertile ART, and fertile control of representative CpGs (A)** ***GRIN2C*** **chr17:70368047, (B)** ***CCDC2*** **chr2:121824853, (C)** ***NDN*** **chr15:21483466, (D)** ***PTPN20B*** **chr10:48448112.**

The graphical representation of methylation distribution of the three study groups at specific CpG sites is shown in Figure 1.

### Differences in candidate gene methylation levels between the three study groups (infertile ART, donor oocyte ART, and fertile control)

As we had three study groups (infertile ART, donor oocyte ART, and fertile control, see ‘Methods’), the most stringent approach to determine whether differences exist is ANOVA followed by Tukey’s *post hoc* HSD test to identify which specific groups are responsible for any significant differences. One-way ANOVA demonstrated that 11 of the 37 CpG sites tested differ between the three groups. When these 11 ANOVA-significant sites were tested for differences between the fertile controls and the two ART groups (infertile and donor oocyte) by Tukey’s *post hoc* HSD test, 7 of the 11 sites differed between the donor oocyte ART group and the fertile control groups and 2 of the sites differed between the infertile group and the fertile controls. Two of the sites differed between the fertile control group and both ART groups (Table 2). Hence, we identified significant differences in approximately 30% of CpGs using ANOVA, and 64% of these differences were attributed to the methylation differences between the donor oocyte ART and fertile control groups.

**Table 2:** **One-way ANOVA and Tukey’s HSD test to assess methylation fraction mean differences in ART (infertile ART and donor oocyte ART) and control groups**

**CpG genomic location** ^**a**^	**Gene**	**Fertile control methylation fraction (mean ± SD)**	**Donor oocyte ART methylation fraction (mean ± SD)**	**Infertile ART methylation fraction (mean ± SD)**	**One-way ANOVA (** ***P*** **-value)** ^**b**^	**Tukey’s HSD test**	
						**Donor oocyte ART** ***vs*** **fertile control (** ***P*** **-value)**	**Female infertile ART** ***vs*** **fertile control (** ***P*** **-value)**
chr12:121824850	*CCDC62*	0.162 ± 0.096	0.101 ± 0.087	0.131 ± 0.078	*0.018*	*<0.01*	ns^c^
chr12:121824853	*CCDC62*	0.165 ± 0.095	0.102 ± 0.084	0.135 ± 0.078	*0.014*	*<0.01*	ns
chr11:122214681	*CRTAM*	0.555 ± 0.069	0.547 ± 0.098	0.556 ± 0.073	0.887	-	-
chr17:30783625	*FLJ10260*	0.586 ± 0.137	0.653 ± 0.107	0.606 ± 0.115	0.106	-	-
chr5:115326619	*FLJ90650*	0.087 ± 0.086	0.119 ± 0.096	0.141 ± 0.114	*0.022*	ns	ns
chr5:115326640	*FLJ90650*	0.100 ± 0.081	0.126 ± 0.113	0.144 ± 0.120	0.094	-	-
chr5:115326626	*FLJ90650*	0.095 ± 0.089	0.147 ± 0.132	0.157 ± 0.131	*0.019*	ns	ns
chr5:115326614	*FLJ90650*	0.098 ± 0.091	0.113 ± 0.112	0.137 ± 0.124	0.175	-	-
chr7:50816700	*GRB10*	0.417 ± 0.079	0.360 ± 0.096	0.452 ± 0.057	*<0.001*	*<0.01*	ns
chr7:50816682	*GRB10*	0.245 ± 0.062	0.205 ± 0.062	0.268 ± 0.064	*<0.001*	*<0.01*	ns
chr7:50816674	*GRB10*	0.269 ± 0.057	0.247 ± 0.064	0.292 ± 0.057	*0.005*	ns	ns
chr7:50816802	*GRB10*	0.620 ± 0.070	0.613 ± 0.078	0.629 ± 0.050	0.529	-	-
chr17:70368047	*GRIN2C*	0.167 ± 0.075	0.091 ± 0.048	0.109 ± 0.065	*<0.001*	*<0.01*	*<0.01*
chr17:70368057	*GRIN2C*	0.108 ± 0.050	0.067 ± 0.033	0.079 ± 0.041	*<0.001*	*<0.01*	*<0.05*
chr11:1975300	*H19*	0.456 ± 0.027	0.443 ± 0.060	0.472 ± 0.055	*0.031*	ns	ns
chr5:131908379	*IL5*	0.710 ± 0.056	0.724 ± 0.053	0.712 ± 0.057	0.605	-	-
chr1:234113562	*LYST*	0.650 ± 0.055	0.649 ± 0.036	0.626 ± 0.068	0.069	**-**	-
chr7:129913072	*MEST*	0.374 ± 0.093	0.372 ± 0.082	0.378 ± 0.094	0.954	-	-
chr7:129913081	*MEST*	0.464 ± 0.105	0.465 ± 0.103	0.477 ± 0.114	0.793	-	-
chr7:129913254	*MEST*	0.278 ± 0.093	0.293 ± 0.084	0.294 ± 0.095	0.635	-	-
chr7:129913259	*MEST*	0.302 ± 0.110	0.326 ± 0.084 ±	0.321 ± 0.102	0.532	-	-
chr15:21483466	*NDN*	0.179 ± 0.076	0.245 ± 0.139	0.212 ± 0.110	*0.041*	*<0.05*	ns
chr15:21483463	*NDN*	0.235 ± 0.101	0.310 ± 0.182	0.275 ± 0.137	0.075	-	-
chr5:140777470	*PCDHGB7*	0.390 ± 0.157	0.389 ± 0.167	0.425 ± 0.145	0.404	-	-
chr5:140777464	*PCDHGB7*	0.312 ± 0.152	0.313 ± 0.156	0.337 ± 0.145	0.627	-	-
chr5:140777418	*PCDHGB7*	0.183 ± 0.103	0.189 ± 0.120	0.213 ± 0.116	0.337	-	-
chr10:48448112	*PTPN20B*	0.199 ± 0.134	0.274 ± 0.148	0.247 ± 0.159	0.094	**-**	-
chr10:48448103	*PTPN20B*	0.239 ± 0.140	0.314 ± 0.176	0.288 ± 0.162	0.115	**-**	-
chr10:48448106	*PTPN20B*	0.215 ± 0.136	0.278 ± 0.176	0.254 ± 0.146	0.191	-	-
chr10:48448108	*PTPN20B*	0.259 ± 0.175	0.351 ± 0.184	0.296 ± 0.161	0.107	**-**	-
chr10:48448115	*PTPN20B*	0.295 ± 0.185	0.385 ± 0.206	0.343 ± 0.181	0.144	-	-
chr15:22620469	*SNRPN*	0.091 ± 0.032	0.098 ± 0.043	0.098 ± 0.054	0.690	-	-
chr15:22644337	*SNRPN*	0.287 ± 0.109	0.299 ± 0.125	0.298 ± 0.123	0.867	-	-
chr15:22644327	*SNRPN*	0.269 ± 0.097	0.274 ± 0.151	0.243 ± 0.109	0.358	-	-
chr17:33179448	*TCF2*	0.545 ± 0.059	0.538 ± 0.077	0.537 ± 0.067	0.804	-	-
chr17:33179450	*TCF2*	0.479 ± 0.067	0.463 ± 0.081	0.476 ± 0.068	0.663	-	-
chr18:27424926	*TTR*	0.660 ± 0.036	0.637 ± 0.049	0.646 ± 0.049	0.094	-	-

^a^Corresponds to genome build 36.1; ^b^values in italics denote significance; ^c^not significant.

### Validation of infertile ART *vs.* fertile control candidate gene methylation differences in an independent population

We performed a one-tailed *t*-test between the infertile and control groups to validate our previous findings demonstrating differential methylation between ART and control groups at 20 CpG sites in 15 genes [30]. Nine of these twenty CpGs showed differential methylation by bisulfite pyrosequencing between the infertile ART and fertile control groups in this validation cohort (Table 3). This represents a 45% validation rate. In the pyrosequencing assay, additional CpGs not present on the original Illumina Infinium 27 K methylation array (Illumina Inc., Sta. Clara, CA, USA) were also interrogated. Of these new CpGs analyzed, nine additional CpGs were found to differ significantly between the infertile and control groups (Table 3). Hence, a total of 18 CpGs (48.65%) showed methylation differences between the infertile ART and the fertile control groups.

**Table 3:** **Difference in methylation fraction means infertile ART and fertile control groups**

**Genomic locations** ^**a**^	**Gene**	**Inclusion in [** 30 **]**	**Infertile ART methylation fraction (mean)**	**Fertile control methylation fraction (mean)**	**Fertile control** ***vs*** **infertile ART (** ***P*** **)** ^**b**^
chr12:121824850	*CCDC62*	Yes	0.131	0.162	*0.030*
chr12:121824853	*CCDC62*	No	0.135	0.165	*0.036*
chr11:122214681	*CRTAM*	Yes	0.556	0.555	0.451
chr17:30783625	*FLJ10260*	Yes	0.606	0.586	0.197
chr5:115326619	*FLJ90650*	Yes	0.141	0.087	*0.003*
chr5:115326640	*FLJ90650*	No	0.144	0.100	*0.015*
chr5:115326626	*FLJ90650*	No	0.157	0.095	*0.003*
chr5:115326614	*FLJ90650*	No	0.137	0.098	*0.035*
chr7:50816700	*GRB10*	No	0.452	0.417	*0.003*
chr7:50816682	*GRB10*	No	0.268	0.245	*0.029*
chr7:50816674	*GRB10*	No	0.292	0.269	*0.016*
chr7:50816802	*GRB10*	No	0.629	0.620	0.199
chr17:70368047	*GRIN2C*	Yes	0.109	0.167	*<0.001*
chr17:70368057	*GRIN2C*	No	0.079	0.108	*<0.001*
chr11:1975300	*H19*	Yes	0.472	0.456	*0.039*
chr5:131908379	*IL5*	Yes	0.712	0.710	0.402
chr1:234113562	*LYST*	Yes	0.626	0.650	*0.025*
chr7:129913072	*MEST*	Yes	0.378	0.374	0.415
chr7:129913081	*MEST*	No	0.477	0.464	0.274
chr7:129913254	*MEST*	Yes	0.294	0.278	0.176
chr7:129913259	*MEST*	No	0.321	0.302	0.178
chr15:21483466	*NDN*	Yes	0.212	0.179	*0.040*
chr15:21483463	*NDN*	Yes	0.275	0.235	*0.046*
chr5:140777470	*PCDHGB7*	Yes	0.425	0.390	0.109
chr5:140777464	*PCDHGB7*	No	0.337	0.312	0.187
chr5:140777418	*PCDHGB7*	Yes	0.213	0.183	0.075
chr10:48448112	*PTPN20B*	Yes	0.247	0.199	*0.045*
chr10:48448103	*PTPN20B*	No	0.288	0.239	*0.047*
chr10:48448106	*PTPN20B*	No	0.254	0.215	0.077
chr10:48448108	*PTPN20B*	No	0.296	0.259	0.121
chr10:48448115	*PTPN20B*	Yes	0.343	0.295	0.084
chr15:22644337	*SNRPN*	Yes	0.298	0.287	0.313
chr15:22644327	*SNRPN*	No	0.243	0.269	0.095
chr15:22620469	*SNRPN*	Yes	0.098	0.091	0.216
chr17:33179448	*TCF2*	Yes	0.537	0.545	0.259
chr17:33179450	*TCF2*	No	0.476	0.479	0.401
chr18:27424926	*TTR*	Yes	0.646	0.660	*0.050*

^a^.Corresponds to genome build 36.1; ^b^values in italics denote significance.

### Gene methylation levels in fertile compared to donor group

As we had no prior knowledge about the direction of results for donor oocyte ART *versus* fertile control groups, we used a two-tailed *t*-test to study these methylation differences. Among the CpGs differing between infertile ART and fertile control groups in the validation (Table 3), 12 CpGs also differed significantly between fertile control and donor oocyte ART groups. These 12 CpGs are shown in Table 4, along with the direction of difference in the Illumina Infinium 27 K array [30] and the pyrosequencing results in the present study for infertile ART and fertile control groups. All significant differences were in the same direction in both the original (array-based profiling) [30] and the validation (bisulfite pyrosequencing) populations. In other words, 67% of the total methylation differences observed between infertile ART and fertile control groups also existed between the donor oocyte ART and fertile control groups.

**Table 4:** **Comparison of methylation fraction mean and** ***P*** **-values at CpGs that differ between ART (infertile and donor oocyte) and fertile control groups**

**CpG genomic locations** ^**a**^	**Gene**	**Fertile control methylation fraction (mean)**	**Infertile ART methylation fraction (mean)**	**Donor oocyte ART methylation fraction (mean)**	**Control > or < infertile in Infinium 27 K/pyro** ^**b**^	**Fertile control** ***vs*** **infertile (** ***P*** **)**	**Fertile control** ***vs*** **donor oocyte ART (** ***P*** **)** ^**c**^
chr12:121824850	*CCDC62*	0.162	0.131	0.101	**>/>**	0.030	0.014
chr12:121824853	*CCDC62*	0.165	0.135	0.102	**>/>**	0.036	0.011
chr5:115326626	*FLJ90650*	0.095	0.157	0.147	**</<**	0.003	0.057
chr7:50816700	*GRB10*	0.417	0.452	0.360	**>/>**	0.004	0.012
chr7:50816682	*GRB10*	0.245	0.268	0.204	**>/>**	0.029	0.013
chr17:70368047	*GRIN2C*	0.167	0.109	0.091	**>/>**	<0.001	<0.001
chr17:70368057	*GRIN2C*	0.108	0.079	0.067	**>/>**	<0.001	0.001
chr15:21483466	*NDN*	0.179	0.212	0.233	**</<**	0.040	0.040
chr15:21483463	*NDN*	0.235	0.275	0.303	**</<**	0.046	0.042
chr10:48448112	*PTPN20B*	0.199	0.247	0.274	**</<**	0.045	0.040
chr10:48448103	*PTPN20B*	0.239	0.288	0.314	**</<**	0.047	0.058
chr18:27424926	*TTR*	0.660	0.646	0.639	**>/>**	0.049	0.034

^a^Corresponds to genome build 36.1; ^b^for CpGs absent in Infinium 27 K array, the direction of difference is considered the same as for other CpGs in the same CpG island; ^c^two-tailed *t*-test.

## Discussion

Our analysis of placental DNA methylation levels at 37 CpG sites in 16 genes demonstrates that there are methylation differences in children conceived *in vivo* compared to children conceived using ART, both in the presence and absence of parental infertility. We observed significant differences in 30% of CpGs between the control, infertile, and donor groups. Furthermore, the majority of these differences (63.63%) could be attributed to the difference between donor oocyte ART and fertile control groups. Additionally, nearly 67% of the differences between ART and control placentas were present in both the infertile and donor groups. Therefore, these results suggest that many of the site-specific DNA methylation differences observed by us and others [1-10] are associated with some aspects of ART clinical and/or laboratory practice(s) rather than some aspect of infertility itself. We also observed that in a subset of genes in Table 2, placental DNA methylation appears to differ between the donor and infertile groups and we are currently validating these findings and investigating the potential role of these changes in the infertility diagnosis. However, it should be noted that in all of these cases, the methylation of the donor oocyte ART group is even more significantly different than the control group when compared to the methylation of the infertile group. This observation strengthens our interpretation of the data that the donor oocyte ART group methylation effects are not due to the infertility, *per se*, but to some aspect of the ART procedures.

Our findings are strengthened by the fact that our donor oocyte ART population had no additional male factor infertility, eliminating the possibility of infertility in either parent as the cause of the observed methylation differences between the donor oocyte ART group and fertile controls. These data are consistent with animal studies that have demonstrated epigenetic changes in domestic cattle (reviewed by [53]), sheep [23], and mice (reviewed by [54]), where no infertility is present. Therefore, the observed changes in animals are attributed to some aspect(s) of the ART procedure(s).

Additionally, we successfully validated placental DNA methylation differences in an independent population for 45% of the CpGs in which we had found differences previously [30]. Given the relatively small differences in mean methylation levels, small sample size (Table 1), and the relatively large variance observed within groups for methylation levels at these specific CpG sites [1,4], we regard the validation of 45% of differences in completely independent populations of ART- and *in vivo*-conceived children as a substantial success rate.

It should be noted that the absolute magnitude of the methylation differences observed between groups is small (Tables 3 and 4) and one might question its clinical significance. However, although the mechanism by which such differences might act is unclear, methylation differences of this magnitude are associated with significant differences in transcript level at multiple genes (Table four and Figures two and three in [1]; Tables one and five in [4]). In the case of the genes interrogated in the present study, several are associated with clinical phenotypes. Necdin (*NDN*), an imprinted gene, is associated with Prader-Willi syndrome [55] and prostate cancer [56]; similarly, *CCDC62* acts as coactivator of the estrogen receptor in prostate cancer cell lines [57]; *GRIN2C*, a member of the NMDA glutamate receptor family, is associated with a number of neurological disorders [58,59]; Laeverin, the gene product of *FLJ90650* gene, has been found to have differential expression in the placentas from patients with pre-eclampsia [60], and methylation levels of *GRB10* are correlated with birth weight [30]. Therefore, we suggest that the small methylation differences observed may prove to be related to a pathophysiologic phenotype in the offspring at a later time and therefore should be considered as important rather than being dismissed as too small to have a clinically relevant significance.

Our study has several limitations. An effect of IVF cycle type (fresh or frozen) on the observed differences cannot be ruled out. Our study group has less than 20% (Table 1) frozen/thawed embryo transfer cycles. Following exclusion of these samples, the analysis of the results of our study remains unchanged (data not shown). An additional limitation is that methylation levels were measured at only a single placental site (directly behind the umbilical cord) and there is some evidence for intra-placental epigenetic variability in humans [4,61]. However, we have measured (see Figures one and three in [4]) DNA methylation levels in five sections of placenta (one tissue sample from each quadrant plus one central sample from directly behind the umbilical cord) from 54 individuals and shown that there is a strong correlation in *IGF2*/*H19* and *IGF2R* methylation between biopsy locations within the same placenta, even though there can be substantial differences between individuals. An additional limitation is that we did not stratify our results by sex because of sample size constraints. It would be interesting to evaluate such methylation differences in male and female placentas, separately; however, the small numbers in the present cohorts, and especially in the donor oocyte ART group, might result in misleading conclusions. Finally, bisulfite conversion and pyrosequencing as a method of determining DNA methylation has limitations, including the possibility of incomplete bisulfite conversion and biased amplification. However, it should be noted that the genes chosen had previously been analyzed by a methylation array, and our study validated many of these previous findings.

## Conclusions

Overall, our data indicate strongly that the majority of the placental DNA methylation differences we observed are associated with ART procedures rather than the diagnosis of infertility *per se*. This is the first study, to our knowledge, that has examined placental DNA methylation following ART in the absence of any known diagnosis of parental infertility in humans. Further studies on larger numbers of patients are needed to evaluate expression level differences, given the much greater inter-individual variability in transcript than in methylation levels (1, 4). Our findings should also direct future study of the underlying mechanism(s) behind the observed differences and focus on the question of which factors might be associated with the observed methylation differences. Elucidating such potentially modifiable factors related to ART-associated methylation differences should guide alterations to clinical and/or laboratory practice protocols, ultimately leading to an improvement in neonatal and long-term outcomes following ART.

## Methods

### Ethics statement

University of Pennsylvania’s Institutional Review Board approved the study (IRB approved protocol no. 804530).

### Samples and sample preparation

Placenta biopsies were collected from *in vivo*-conceived controls (fertile control group) and ART children conceived following fertilization of oocytes from two groups: 1) autologous oocytes with infertility in one or both parents (infertile ART group) and 2) donor oocytes (obtained from young fertile donors) without male factor infertility (donor oocyte ART group). Genomic DNA was prepared as described [1,4]. All IVF cycles were performed at a single center and the clinical and laboratory procedures are uniform for all the samples. The fertile control group was conceived without medical assistance (infertility medications or treatments) and the parents had no prior history of infertility. All of the biopsies used in the present study were taken from the same location in each placenta, directly behind the cord on the maternal side. Eighty-eight ART placental samples (22 in the donor oocyte ART group and 66 in the female infertile ART group) and 49 controls were analyzed for all CpGs. Mean maternal age (the maternal age of recipient was included in case of the donor group), mean gestational age, and mean birth weight, as well as sex of controls and ART children, are also described in Table 1. All donor oocytes were obtained from young healthy women between the ages 21 and 33.

### Selection of candidate genes

We selected candidate genes for validation of DNA methylation differences between ART and controls from cord blood and placenta samples assayed on an Illumina Human Methylation27 Bead-Chip array that was analyzed in a previous study [30]. The candidate genes whose methylation differed significantly between ART (*n* = 24) and control (*n* = 24) individuals were selected based on the following: 1) differential methylation of two or more CpG sites within the same gene (*P* < 0.05 at each, two-tailed *t*-test); 2) absolute magnitude of mean methylation differences, those with differences >0.07 were retained for further consideration; 3) ability to design high-quality pyrosequencing assays. Using these criteria, seven genes (*GRIN2C*, *PCDHGB7*, *PTPN20B*, *MEST*, *TCF2*, *FLJ10260*, *NDN*) were selected (additional genes fulfilling these criteria were available; however, high-quality pyrosequencing assays could not be designed or the assays failed). We considered further those candidate genes represented by only a single CpG on the array or for which only a single CpG was significant but that the mean absolute magnitude of difference was >0.07. Under these criteria, six additional genes (*CRTAM*, *TTR*, *SNRPN*, *IL5*, *LYST*, *FLJ90650*) were selected. The imprinted genes *H19* and *GRB10* were also selected because we had observed differences for multiple CpGs at both loci in previous studies [1,30], and 7 of the 16 *H19* CpGs and 8 of the 12 *GRB10* CpGs differed significantly on the array. Lastly, *CCDC62* was selected because of the large mean difference between ART and control groups for both CpGs on the array (−0.079 and −0.062). Even though these differences did not reach statistical significance, the very large variance and absolute magnitude of difference in maximum/minimum beta values (>0.4) suggested that the significance might be reached on examining a larger number of individuals.

It is a common observation (for example, Figure two in [1]; Figure two in [4]; Figures one and two in [62]) that within a CpG island, methylation at one site is highly correlated with methylation at other sites within an individual. Hence, we selected one CpG site per CpG island for methylation analysis, except in the cases where multiple CpG sites could be analyzed in a single pyrosequencing assay. Hence, a total of 22 CpGs in 16 genes were selected for designing of pyrosequencing assays.

### Design and performance of pyrosequencing assays

The designing of pyrosequencing assays for the 22 CpGs thus selected resulted in inclusion of 15 additional CpGs that were in close proximity to the selected CpGs. The genomic location of the 37 CpGs interrogated in the 16 genes by the bisulfite pyrosequencing assays and the primer sequences used are in Additional file 1.

### Statistical analysis

One-way ANOVA was used to estimate differences between the three groups (infertile ART, donor oocyte ART, and fertile control). The significant results of ANOVA were analyzed using Tukey’s HSD test. One-tailed *t*-tests were used in comparing mean methylation levels between infertile ART and fertile control groups for the validation of our previous array findings [1,30] as we had a prior expectation for the direction of change and the use of one-sided tests is an accepted statistical procedure in validation studies [63]. Two-tailed *t*-tests were used for comparing fertile control and donor oocyte ART groups. A *P*-value of <0.05 was considered significant, without correction for multiple testing, because we were validating results for candidate genes shown previously to differ between ART- and *in vivo*-conceived children.

## Additional file

Additional file 1:
**Primer sequences for the pyrosequencing assays.**
