# Supplementary material for: DNA methylation differences between in vitro- and in vivo-conceived children are associated with ART procedures rather than infertility
Source: Clin Epigenetics. 2015 Apr 8;7(1):41. doi: 10.1186/s13148-015-0071-7 (PMC4404660; doi:10.1186/s13148-015-0071-7)
Supplement: Additional file 1: — Primer sequences for the pyrosequencing assays. [file 13148_2015_71_MOESM1_ESM.pdf]

Additional file1: Primer sequences for the pyrosequencing assays

Genomic locations[a]	Gene	Primer sequences[b]	Biotin labeled primer
chr12:121824850	***CCDC62***	F: 5’ ATTGGGGGTTTTTAAGTGGTTTAG 3’<br>R: 5’AACAACCTTTATTAACTTCTTCATTCTCTA 3’<br>S: 5’ ATTCTTTCAAACCTTCCA 3’	Forward
chr12:121824853	***CCDC62***		
chr11:122214681	***CRTAM***	F: 5’ ATGTTTAAATTATGTATTTGAGGGATGTT 3’<br>R: 5’ AAAACCCACAAAAACACACACT 3’<br>S: 5’ ATGTATTTGAGGGATGTTA 3’	Reverse
chr7:96488107	***DLX5***	F: 5’ GGAATTGATTGAGTTGGTTGTATTTG 3’<br>R: 5’ CTCAACCACCACCCTCATA 3’<br>S: 5’ TATTTGTGTATTAGGATGTAGAGT 3’	Reverse
chr17:30783625	***FLJ10260***	F: 5’ TGGTTATTTGGTGTGTATATAATGTAAA 3’<br>R: 5’ ATAACTTCACTTCAACCTCCCATTA 3’<br>S: 5’ AGATTAGTTTGAATGGTTGTA 3’	Reverse
chr5:115326619	***FLJ90650***	F: 5’ GTAGTTTTGGAAGAGGTTATGT 3’<br>R: 5’ ATCTTCCCCTCCCCTCAA 3’<br>S: 5’ TTTGGAAGAGGTTATGTA 3’	Reverse
chr5:115326640	***FLJ90650***		
chr5:115326626	***FLJ90650***		
chr5:115326614	***FLJ90650***		
chr11:22646220	***GAS2***	F: 5’ AGAGTTTGGAGTAAAGAAGTATAGG 3’<br>R: 5’ ACAAAAACACCCATTTAACATCC 3’<br>S: 5’ TTGTGAATTTTAGGGGAT 3’	Reverse
chr7:50816700	***GRB10***	F: 5’ ATTGTTTTTTTAAGTTTGAAAGGTAG 3’<br>R: 5’ CAAAAAACTCCAAACAACACC 3’<br>S: 5’ TTTTTTTTAAGTTTGAAAGGTAG 3’	Reverse
chr7:50816682	***GRB10***		
chr7:50816674	***GRB10***		
chr7:50816802	***GRB10***	F: 5’ TTTTTGTTAGGGTGTTGGG 3’<br>R: 5’ CCCTCACTAAACTAAAAAAAAAAACTTTAC 3’<br>S: 5’ GTATATAGGTGGTTATG 3’	Reverse
chr17:70368047	***GRIN2C***	F: 5’ GGGGTTAGGGATTGAATTAAGA 3’<br>R: 5’ CCCTAATACCCACCCCATTC 3’<br>S: 5’ GGTTAGGGATTGAATTAAGATA 3’	Reverse
chr17:70368057	***GRIN2C***		
chr11:1975300	***H19***	F: 5’ AAGAAGGGGGAGTTAGGTATTTA 3’<br>R: 5’ AAACCTCCTTCTTTCAAAAAACTAA 3’<br>S: 5’ GTGGTAGTTGGTTGGA 3’	Reverse
chr5:53787676	***HSPB3***	F: 5’ GGGTGAAGTTTTTTGAGATAAAAT 3’<br>R: 5’ AAAACAAATCCCACTTTCAAATC 3’<br>S: 5’ GTGTTTATTTATTTTGGT 3’	Reverse
chr1:157245543	***IFI16***	F: 5’ TGTTGAGTAGAGGATTAAATAGATTT 3’<br>R: 5’ ACTATCCTCTTAACCATAACTAAATTACT 3’<br>S: 5’ TTAGTGTATTTTTTAGGAT 3’	Reverse
chr1:157245550	***IFI16***		
chr5:131908379	***IL5***	F: 5’ GGTTTGAATATATATGATGGAGGTGTATGT 3’<br>R: 5’ TATATCTACCTCCCAACTACCTCTACT 3’<br>S: 5’ ATGTTGTAGTTTAGAAGTTTGAT 3’	Reverse
chr1:234113562	***LYST***	F: 5’ AGTTAAAAGGTTATTGGGATGGT 3’<br>R: 5’ AACTTAATTTACCAAATTCCCCATATAA 3’<br>S: 5’ ATTGGGATGGTTTTTTAGA 3’	Reverse
chr7:129913072	***MEST***	F: 5’ TTGGTTTTGTTGAGTATGTGATG 3’<br>R: 5’ATAAAAACCAAAAAAATAACATTTAATAA 3’<br>S: 5’ AAATTAGGGGAAGGG 3’	Reverse
chr7:129913081	***MEST***		
chr7:129913254	***MEST***	F: 5’ GAAATTAGGGGAAGGGTTGAAA 3’<br>R: 5’ CCTTCTCCCTACCAAAC 3’<br>S: 5’ TATTTTATATTTTTTGTAATAGGTGG 3’	Reverse
chr7:129913259	***MEST***		
chr15:21483466	***NDN***	F: 5’ GGGTTTAGAGGAGGG 3’<br>R: 5’ CAAAAACCCTACCCTTACCAA 3’<br>S: 5’ AGATTTTTATTTTGTTTTGATATG 3’	Reverse
chr15:21483463	***NDN***		
chr5:140777470	***PCDHGB7***	F: 5’ GGTAATTTGGTGTGTTGGGTAAG 3’	Reverse

chr5:140777464	***PCDHGB7***	R: 5’ ATCCCTCAACCTCTAACCTA 3’<br>S: 5’ GTTGGGTAAGGTTTG 3’	
chr5:140777418	***PCDHGB7***	F: 5’ GGTGTGTTGGGTAAGGTTTG 3’<br>R: 5’ CCCTCAACCTCTAACCTAAAAT 3’<br>S: 5’ CAACCTCTAACCTAAAATTCCCT 3’	Forward
chr14:23633270	***PCK2***	F: 5’ AAGAGTGGATTTAGTTTTTTAATGG 3’<br>R: 5’ AAACCTAATAAAACTAACACTAACTT 3’<br>S: 5’ AGGGAGTTGGTTTGT 3’	Reverse
chr14:23633275	***PCK2***		
chr10:48448112	***PTPN20B***	F: 5’ GTTTGGGAGAGGGGGATT 3’<br>R: 5’ CCCCAACCACCTTTCCTCA 3’<br>S: 5’ CAAAACACTAAAAACCCAT 3’	Forward
chr10:48448103	***PTPN20B***		
chr10:48448106	***PTPN20B***		
chr10:48448108	***PTPN20B***		
chr10:48448115	***PTPN20B***		
chr15:22644337	***SNRPN***	F: 5’ TGAGGTTGGAGGATTTAGGTTGTG 3’<br>R: 5’ ATATTTCCCTACACATCACTCT 3’<br>S: 5’ GATTTAGGTTGTGAGGTA 3’	Reverse
chr15:22644327	***SNRPN***		
chr15:22620469	***SNRPN***	F: 5’ TGGTTGAAAAGGAGAGGGTTAGA 3’<br>R: 5’ ATTAATTCCTTATACACCTTCCACAA 3’<br>S: 5’ AGGTTTAAAGAAGTTATGATAG 3’	Reverse
chr17:33179448	***TCF2***	F: 5’ ATTTAATTTTTGGATTTGTTAAGTTAGTGT 3’<br>R: 5’ CCCTTCCTAAATAATCAATTTCTCTT 3’<br>S: 5’ ATTTGTTAAGTTAGTGTTTTGTA 3’	Reverse
chr17:33179450	***TCF2***		
chr18:27424926	***TTR***	F: 5’ GTTTTGGTTTTTTAAAGTGTTGGGATTA 3’<br>R: 5’ CAACTATCAACCTTATTCCTACATACCC 3’<br>S: 5’ TTTTTTAAAGTGTTGGGATTAT 3’	Reverse

a. Corresponds to genome build 36.1
b. F forward ; R reverse ; S sequencing
